# Mass fingerprinting and electrophysiological analysis of the venom from the scorpion *Centruroides hirsutipalpus* (Scorpiones: Buthidae)

**DOI:** 10.1186/s40409-018-0154-y

**Published:** 2018-07-03

**Authors:** Laura L. Valdez-Velázquez, Timoteo Olamendi-Portugal, Rita Restano-Cassulini, Fernando Z. Zamudio, Lourival D. Possani

**Affiliations:** 1 Facultad de Ciencias Químicas, Universidad de Colima, Coquimatlan, Colima, Mexico; 2 Instituto de Biotecnología, Universidad Nacional Autónoma de México, Avenida Universidad 2001, Colonia Chamilpa, apartado postal, 510-3 Cuernavaca, Morelos Mexico

**Keywords:** *Centruroides*, Electrophysiology, Mass fingerprinting, Scorpion venom

## Abstract

**Background:**

*Centruroides hirsutipalpus*, of the family Buthidae, is a scorpion endemic to the Western Pacific region of Mexico. Although medically important, its venom has not yet been studied. Therefore, this communication aims to identify their venom components and possible functions.

**Methods:**

Fingerprinting mass analysis of the soluble venom from this scorpion was achieved by high-performance liquid chromatography and electrospray mass spectrometry. Furthermore, the soluble venom and its toxic effects were evaluated extensively via electrophysiological assays in HEK cells expressing human voltage-gated Na^+^ channels (hNav 1.1 to Nav1.6), CHO cells expressing hNav 1.7, potassium channel hERG 1 (Ether-à-go-go-related-gene) and the human K^+^-channel hKv1.1.

**Results:**

The separation of soluble venom produced 60 fractions from which 83 distinct components were identified. The molecular mass distribution of these components varies from 340 to 21,120 Da. Most of the peptides have a molecular weight between 7001 and 8000 Da (46% components), a range that usually corresponds to peptides known to affect Na^+^ channels. Peptides with molecular masses from 3000 to 5000 Da (28% of the components) were identified within the range corresponding to K^+^-channel blocking toxins. Two peptides were obtained in pure format and completely sequenced: one with 29 amino acids, showing sequence similarity to an “orphan peptide” of *C. limpidus*, and the other with 65 amino acid residues shown to be an arthropod toxin (lethal to crustaceans and toxic to crickets). The electrophysiological results of the whole soluble venom show a beta type modification of the currents of channels Nav1.1, Nav1.2 and Nav1.6. The main effect observed in channels hERG and hKv 1.1 was a reduction of the currents.

**Conclusion:**

The venom contains more than 83 distinct components, among which are peptides that affect the function of human Na^+^-channels and K^+^-channels. Two new complete amino acid sequences were determined: one an arthropod toxin, the other a peptide of unknown function.

## Background

Toxins from microorganisms, plants and animals are usually produced by highly specialized systems of exocrine cells or are synthesized in specific tissues of the organisms [1]. The toxins of arachnid origin, especially those from scorpions, are produced in a pair of glands located in the last segment of the metasoma, called the telson. A stinger situated at the telson tip serves as the inoculating device. The type of venom produced depends on the scorpion species, but usually the venom is neurotoxic and affects the central or peripheral nervous system of vertebrates and arthropods. The main effect is a modification of ion channel function of both excitable and non-excitable cells, often producing paralysis of the prey [2]. All scorpions are poisonous, but only a few species are extremely dangerous to humans.

In Mexico scorpions of the genus *Centruroides* can be lethal to humans. LD_50_ values in mice by subcutaneous injection can be as low as 0.075 μg/g body weight [3]. Originally, eight scorpion species in the country were clearly identified and reported as dangerous to humans: *C. limpidus, C. noxius, C. infamatus, C. elegans, C. tecomanus, C. pallidiceps, C. sculpturatus* and *C. suffusus* [4]. Recently, in the state of Colima, a very toxic species belonging to the family Buthidae was identified: *Centruroides hirsutipalpus* [5], on which no information is available concerning their venom components, structure and function. It is one of the thirteen species of dangerous scorpions now known to occur in Mexico [6].

The scorpion *C. hirsutipalpus* is endemic to this Western Pacific region of Mexico. This specie is morphologically and geographically related to two other species of “striped scorpions”: *C. elegans* and *C. tecomanus* [5]. In this communication, we report the separation of its venom by chromatographic methods and identification of their molecular masses by mass spectrometry. The toxicity of the soluble venom was evaluated by electrophysiological assays and is herein reported for the first time. In addition, two interesting peptides obtained in homogeneous form were fully sequenced by Edman degradation, one with a sequence similar to an “orphan peptide” from the scorpion *C. limpidus*, while the other was shown to be an arthropod toxin lethal to crustaceans and toxic to crickets.

## Methods

### Biological material

The scorpion specimens were collected in the in the community of Minatitlan in the Mexican state of Colima in June 2016 (latitude 19°23′01.73´´N; longitude 104°03′35.19´´O; elevation 703 m above sea level), under an official collection permit from SEMARNAT (SGPA/DGVS/12063/15 granted to Laura Valdez). The animals were kept in captivity (standard conditions of temperature, light and dark periods, water ad libitum and fed crickets) for 15 days. The venom was extracted from 25 scorpions via electrical stimulation (15 V shock applied to the animals), dissolved in water, centrifuged at 14,000 *g* for 15 min; then the supernatant was immediately lyophilized and kept at − 20 °C until use.

### Chromatographic separation of soluble venom and mass fingerprint analysis

The fractionation of soluble venom was performed by high performance liquid chromatography (HPLC) on an analytical C18 reverse-phase column (dimensions of 4.6 × 250 mm) obtained from Grace Vydac (USA). Samples of lyophilized venom (0.75 mg protein content) were dissolved in 500 μL of solvent A [0.12% trifluoroacetic acid (TFA) in water] and applied to the column. Elution was obtained by running a linear gradient with solvent A (0.12% trifluoroacetic acid in water) to 60% of solvent B (0.10% TFA in acetonitrile), for 60 min at a flow rate of 1 mL/min. The protein content of the venom and fractions was estimated based on absorbance at λ = 280 nm, assuming that one unit of absorbance is equal to 1 mg/mL. The fractions were collected manually by monitoring the absorbance at 230 nm and then dried in a Savant Speed Vac SC210A apparatus (USA). The various fractions obtained from the HPLC separation were dissolved in 50% acetonitrile containing 1% acetic acid to achieve a final concentration of approximately 0.1 to 0.5 mg/mL. This concentration was estimated based on the area under the curve of the various subfractions obtained from the chromatogram of the HPLC separation. All samples were analyzed using an LCQ Fleet mass spectrometer (Thermo Finnigan, USA).

### Primary structure determination

Homogeneous components were submitted to Edman degradation using a PPSQ-31A protein sequencer from the company Shimadzu Scientific Instruments, Inc. (USA). When needed, the peptide was reduced and carboxymethylated for confirmation of the cysteine residues. Additionally, the alkylated peptide was enzymatically digested with Asp-N endopeptidase (Roche, Germany) for completion of the primary sequence. The latter step was performed under the same conditions already described for other toxic peptides, according to Olamendi-Portugal et al. [7].

### Lethality tests

Experiments showing that this species is dangerous to humans were reported earlier by our group [6]. Herein we assayed the new purified and sequenced peptides in two additional animals: freshwater crayfish (*Cambarellus montezume* ssp.) and crickets (*Acheta* sp.), as described previously [8].

### Electrophysiological analyses

Electrophysiological analyses of the soluble venom were performed using HEK cells expressing human voltage-gated Na^+^ channels (hNav 1.1 to Nav1.6) and CHO cells expressing hNav 1.7 and potassium channels hERG 1 (Ether-à-go-go-related-gene) and hKv1.1. All cells were maintained in Dulbecco’s modified Eagle medium (DMEM) (Sigma, Mexico) supplemented with 10% fetal bovine serum (FBS) (Byproductos, Mexico), at 37 °C with 5% CO_2._ Antibiotic G418 at 400 μg/mL concentration was added to the medium. Cells expressing hNavs sodium channels and plasmid for hERG were kindly donated by Professor Enzo Wanke from the University of Milano Bicocca, Milan, Italy. The extracellular solution expressed in mM was: 130 NaCl, 5 KCl, 2 CaCl_2_, 2 MgCl_2_, 10 HEPES and 5 glucose, at pH 7.3 adjusted with NaOH. For hERG potassium currents, extracellular solution had 40 mM KCl and 95 mM NaCl. For sodium channels records, intracellular solution contained in mM: 105 CsF, 27 CsCl, 5 NaCl, 2 MgCl_2_, 10 EGTA, 10 HEPES, pH 7.3 adjusted with CsOH. For potassium channel records, the intracellular solution expressed in mM was: K-aspartate 130, 10 NaCl, 2 MgCl_2_, 10 HEPES and 10 EGTA, at pH 7.3 adjusted with NaOH.

Sodium currents were elicited by means of a step depolarization for 100 ms, from − 120 to 40 mV with 10 mV increase, followed by a 50 ms step at full-activation potential (− 10 mV or − 30 mV in the case hNav 1.5 channels). The holding potential was set at − 120 mV and a short strong depolarization pre-pulse (5 ms at 50 mV) was applied 50 ms before the depolarization steps. Potassium currents of the type hKv 1.1 were elicited by a step depolarization at 60 mV for 200 ms, followed by a step at − 50 mV for 200 ms. Pulses were applied every 6 s. Currents for the hERG channel were elicited as tail currents by means of a depolarization step at 60 mV for 500 ms followed by a repolarization step at − 120 mV for 500 ms. Pulses were applied every 6 s. Currents were registered by means of the amplifier MultiClamp 700 B along with the analog-digital converter Digidata 1440A and software pCalmp10 (Molecular Devices, USA). Data were analyzed with the software Clampfit10 (Molecular Devices) and Origin 7 (OriginLab, USA).

## Results

### HPLC separation and mass fingerprinting

**Fig. 1:**
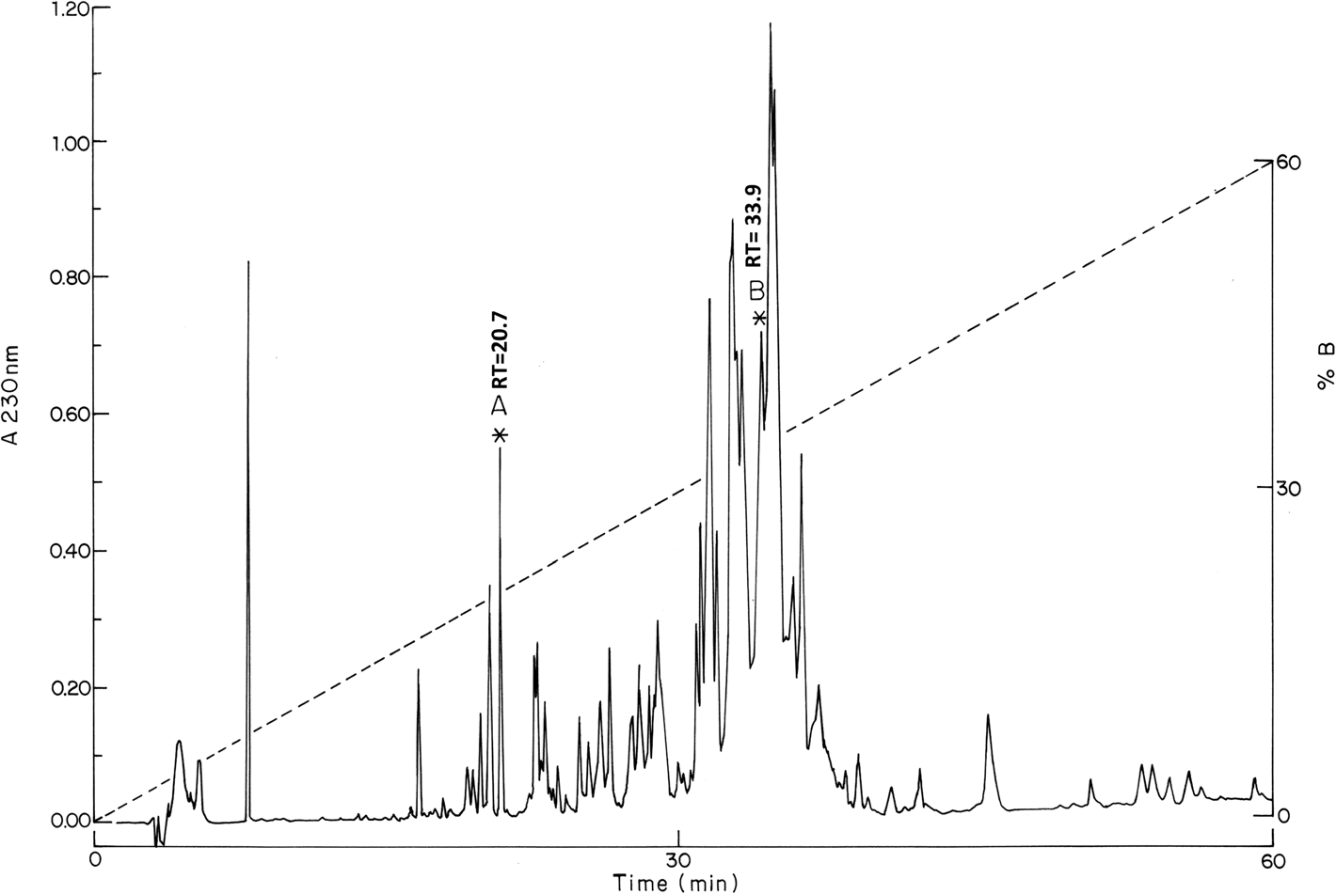
Separation by HPLC of the venom of *Centruroides hirsutipalpus*. The soluble part of scorpion venom (0.75 mg of protein) was passed through a reverse phase C18 column and separated with a linear gradient of a solvent A (0.12% trifluoroacetic acid in water) to 60% of solvent B (0.10% TFA in acetonitrile), for 60 min

Separation of soluble venom by HPLC (Fig. 1) revealed more than 56 clear chromatographic peaks, which were collected in 60 distinct fractions. From these, at least 83 different components were identified by mass spectrometry, with molecular weights varying from 340 to 21,120 Da. The results obtained are shown in Table 1. The most abundant components found were eluted at the following retention times (RT): 20.7, 31.4, 32.5, 32.8, 33.1, 33.9, 34.0, 34.4, 34.7 and 36.1 min and are shown in bold. The components of RT 20.7 and 33.9 min were sequenced as described below. All other components have molecular weights within the range of the known Na^+^-channel toxins. A few components (total 6) were not identified, either due to their chemical compositions or complexity (several components in the fractions, impeding individual bona fide identification). Please note that a few components with identical masses separated at different eluting times of the HPLC.

**Table 1:** Determination of molecular weights (MW) by mass spectrometry of the venom components from *C. hirsutipalpus*

RT (min)	MW (Da)	RT (min)	MW (Da)
4.4	ND	29.8	4296.99
5.1	ND	30.0	4296.9, 6937.5
5.4	ND	30.4	6461
7.8	535.3	30.6	7428.11
16.6	2611.41, 2723.94,2837	30.8	7373.35
17.4	492	31.4	**7214, 5163.79, 7501.78**
17.8	340	31.7	7422.98
18.2	1288	32.5	**7590.77, 7157**
19.1	3665.55, 4097.56, 2611.8	32.8	**7591.17**
19.3	3881.16, 3994.3	33.1	**7380.56, 7051.53, 7591**
19.7	4325.88	33.9	**7051.53**
20.2	764.67, 4325.78	34.0	**7052.03, 7751.45, 7791.7**
20.7	**3421.84**	34.4	**7791.31, 7299, 7051.5**
21.7	943.4	34.7	**7791.8, 7462, 7276.5, 9142**
22.2	2112.6	35.2	7275.29
22.5	4123.9	35.7	7287.28
22.7	4212.45	36.1	**7169.63, 7098.16**
23.0	4039.7, 4189.39, 4212	36.5	7431, 7170.32, 7536.07
23.4	4170.16, 4281.63	36.8	7170.19, 7431.25
23.6	3982.46	37.4	7433.04, 7170.2
24.1	3994.67, 1742	38.0	7432.41, 7475.39
24.7	3988.18	38.3	7013.31
25.2	4001.86	38.9	5931, 7600.73
25.8	7765,4237.23, 4084.95	39.4	4698.84
26.3	4775.83	40.6	7771.3
27.4	4156.12, 6963.27	42.0	13,476.8
27.9	7439.89, 6979.39	45.5	21,120
28.3	7312.05	51.0	ND
28.7	6587.35, 7293	53.9	ND
29.0	6587	55.7	ND

Bold numbers means more abundants components

**Fig. 2:**
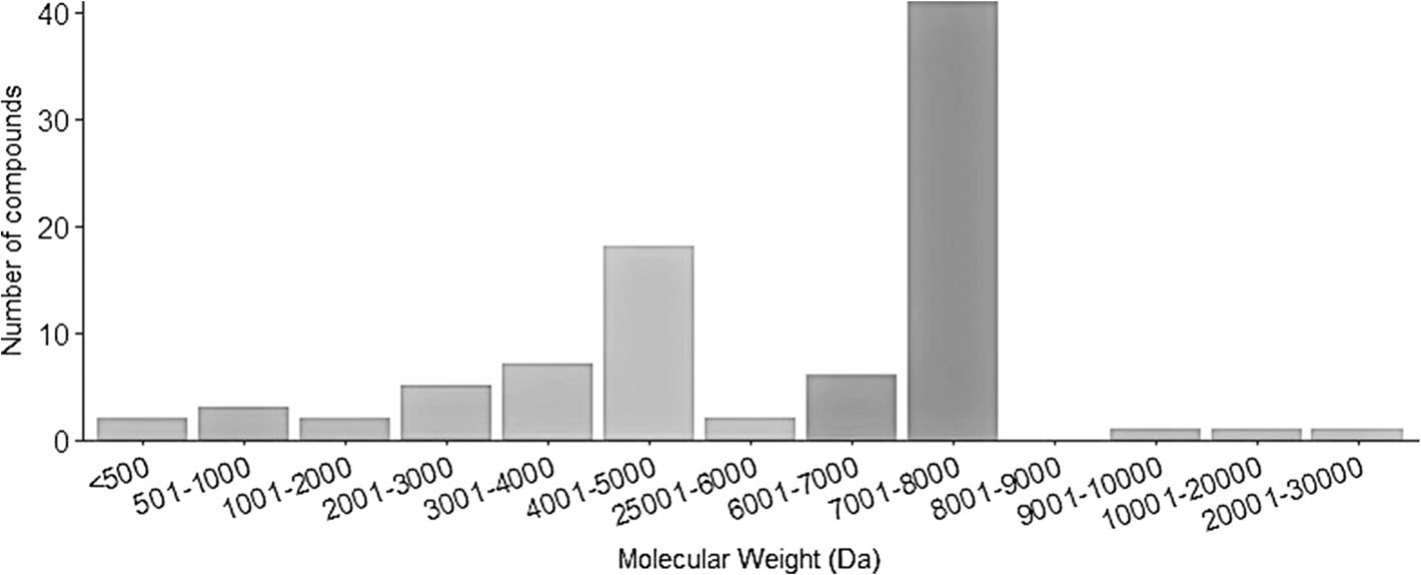
Fingerprinting of soluble venom. The histogram shows the frequency distribution of molecular weights (MW), in Da, for the 89 peptide masses determined (from which 83 were distinct), present in the venom of *Centruroides hirsutipalpus*, clustered within different intervals of molecular weights (1000 Da apart from each other). The MWs were obtained using the mass spectrometer LCQ Fleet

The molecular mass distribution of the venom components found in the 60 fractions are displayed in Fig. 2 clustered within different intervals of molecular weights, mostly 1000 Da apart from each other. Four groups of components with distinct molecular masses were found: < 500–1000 Da (5.6%), 1001–5000 Da (36%), 5001–9000 Da (55%) and 9001–30,000 Da (3.4%). The majority of peptides have a molecular weight of 4001 to 5000 Da or from 7001 to 8000 Da, ranges that usually correspond to peptides known to affect K+ channels and Na + channels, respectively.

### Primary structure determination

**Fig. 3:**
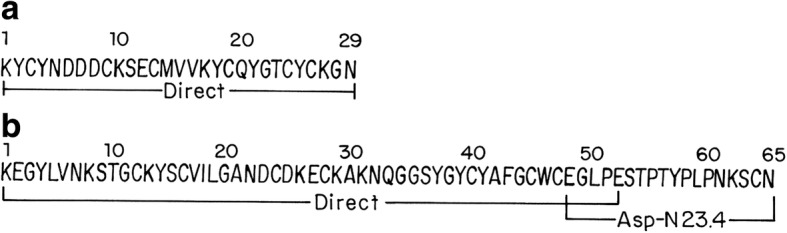
Primary structure of purified peptides. (**a**) Peptide with molecular weight 3421.84 Da, containing 29 amino acids. Captioned “Direct” means sequence obtained directly by automatic Edman degradation. (**b**) Peptide of 7051.53 Da containing 65 amino acids and sequenced directly by Edman degradation, and completed by sequencing the peptide “Asp N 23.4” obtained after enzymatic digestion of a reduced and alkylated sample

Subfractions eluted at 20.7 min and 33.9 min, labeled A and B, respectively, in Fig. 1, were shown to be homogeneous by mass spectrometry and were used for determination of their primary structures. Figure 3 shows the primary structure of these peptides. Peptide A was sequenced automatically by Edman degradation. This peptide has a molecular weight of 3421.84 Da and contains 29 amino acids, among which are six cysteines that form three disulfide bonds. After reduction and alkylation, the full amino acid sequence was obtained. Peptide B has molecular weight of 7051.53 Da and contains 65 amino acids, including eight cysteines that form four disulfide bonds. The first 52 amino acids at the N-terminal region were identified directly by Edman degradation. An overlapping segment at the C-terminal section, residues 48 to 65, was identified after reduction, carboxymethylation and digestion with endopeptidase Asp N. This toxin fragment was separated by HPLC eluting at 23.4 min (data not shown).

The two new peptides sequenced and reported herein are very interesting peptides as will be discussed later. One was 97% identical to CllNtx, a peptide of undetermined function [9], whereas the other was shown to be an arthropod toxin. Each of four crayfish and four crickets was injected with 50 μg of this peptide. All animals were paralyzed immediately after injection. The effect was more pronounced in crayfish, which became paralyzed for at least 8 h. One of them died within the first 24 h; the other three recovered. The crickets were transiently paralyzed, surviving the injection.

### Electrophysiological analyses

The effects of the C. hirsutipalpus-soluble venoms were analyzed at 20 μg/mL concentration, on seven subtypes of human sodium channels (hNav 1.1–1.7) and two sub-types of K+ channels (hKv1.1 and hERG).

**Fig. 4:**
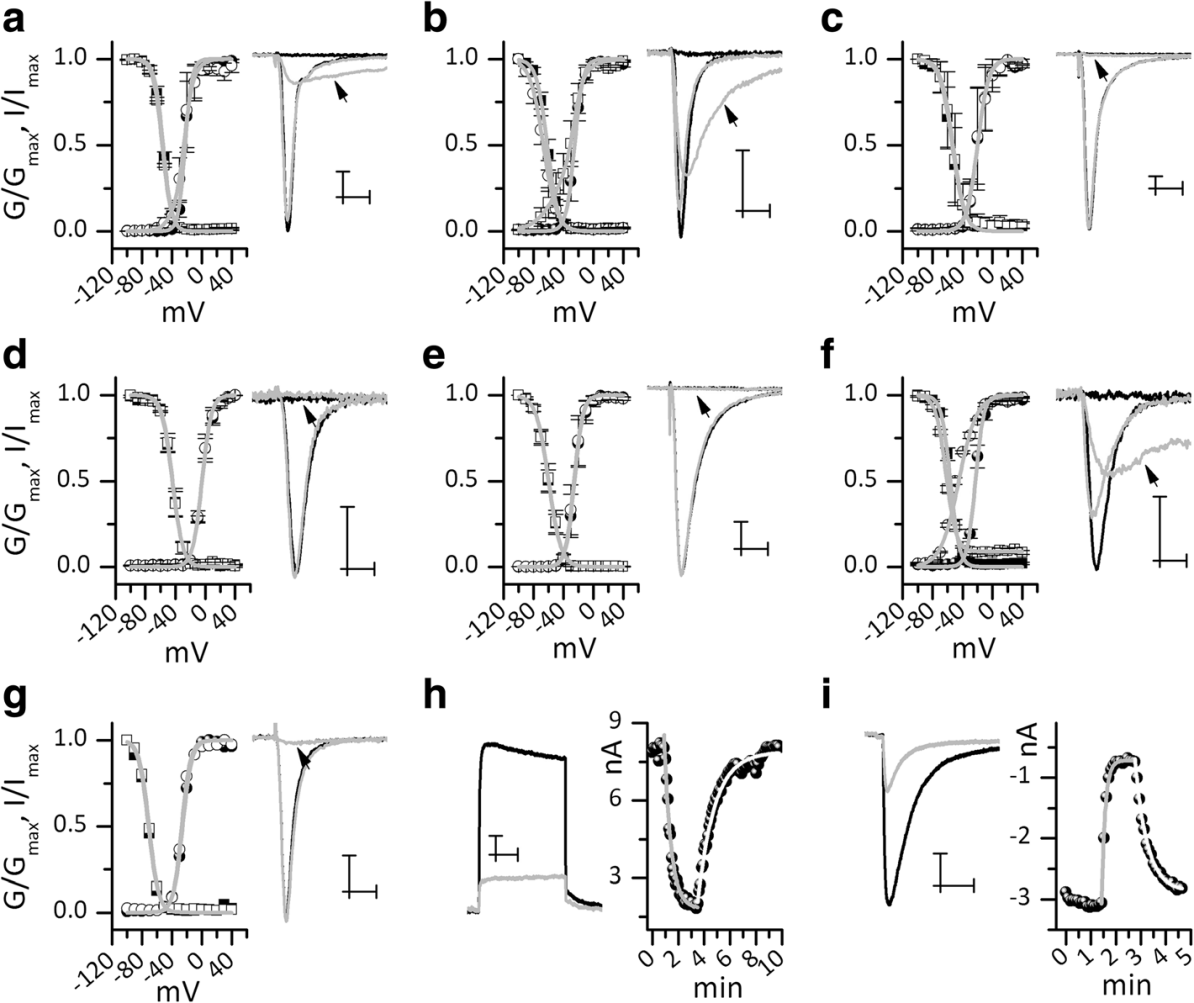
Electrophysiological characterization of soluble venom. The effect of soluble *Centruroides hirsutipalpus* venom on sodium and potassium ion channels. From panels A to G: sodium currents of hNav 1.1 to 1.7 channels, respectively. On the right side of each panel are represented the current traces elicited with stimulus at − 10 mv (maximal current) and at − 50 mv (sub-threshold stimulus). Black lines are for control and grey lines are for venom application. In (**a**) hNav 1.1, (**b**) hNav 1.2 and (**f**) hNav 1.6, venom produced current at − 50 mV (arrow) and reduction of the maximal current. The (**c**) hNav 1.3, (**d**) hNav 1.4, (**e**) hNav 1.5 and (**g**) hNav 1.7 were insensitive to the venom. In each panel, on the left, are represented the activation (circlet) and the inactivation (squares) curves. Full symbols are for the control and empty symbols are for the venom. Grey line are the best fit from a Boltzmann equation or the sum of two Boltzmann equations. In (**a**), (**b**) and (**f**) venom moves activation curve to more negative potentials. (**h**) Venom produced current reduction on hKv 1.1 channels. The left side shows current in control (black line) and after venom application (grey line). On the right, current values were plotted against time and data were fitted with a first-order exponential equation. Grey line is the best fit of the on-kinetic data and light gray line is the best fit of the off-kinetic data. (**i**) Venom produced current reduction on hERG1 channels. The left side shows current in control (black line) and after venom application (grey line). On the right, current values were plotted against time and data were fitted with a first-order exponential equation. Grey line is the best fit of the on-kinetic data and light gray line is the best fit of the off-kinetic data. Scaler is 1 ms and 1 nA in panels (**a**), (**b**), (**c**), (**e**), (**g**); 1 ms and 0.5 nA in panels (**d**), (**f**); 50 ms and 1 nA in panels (**h**) and (**i**)

Venom application to the sodium channel produced modification in the voltage dependence of the activation process. This effect, which is typical of scorpion beta toxins, was evident on hNav 1.1, hNav 1.2 and on hNav 1.6 subtypes (Fig. 4, panels a, b and f). In these channels, especially in hNav 1.6, venom induced activation at more negative potential and reduction of peak current (Fig. 4, panels a, b and f). By contrast, hNav 1.3, hNav 1.4, hNav 1.5 and hNav 1.7 were insensitive to venom at the used concentration (Fig. 4, panels c, d, e and g). None of the sodium channels in analysis showed any change in the inactivation process after venom application (Fig. 4, panels a-g).

Venom was also able to reduce currents of the voltage-gated potassium channels hKv 1.1 and hERG, when applied at 20 μg/mL concentration. In both cases, venom action was reversible (Fig. 4, panels h and i, respectively). Current values were plotted against time; then data were fitted with an exponential equation to extrapolate the on- and off-kinetic time constants (Ʈon and Ʈoff, respectively). In Fig. 4, panel (**h**) and (**i**) the grey line is the best fit for on-kinetic data and light grey line is the best fit for off-kinetic data fitting. The resulting time constants were for hERG1: Ʈon 9.4 s and Ʈoff 27.4 s; for hKv 1.1: Ʈon 32 s and Ʈoff 77 s. Based on the equation:

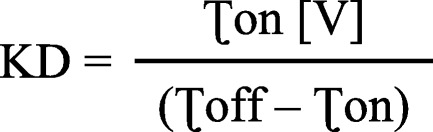



the apparent venom concentration that produced a half effect (KD) was estimated at 10 μg/mL for hKv 1.1 and 14 μg/mL for hHERG1 (venom concentration [V] was 20 μg/mL). In Table 2, we summarize the data obtained for activation and inactivation of the channels, under the effect of the whole soluble venom.

**Table 2:** Activation and inactivation V1/2 values

Sodium channel	V_1/2_ activation (mV)		V_1/2_ inactivation (mV)	
	Control	Venom	Control	Venom
hNav 1.1	−22.4 ± 0.5	−26.5 ± 3.4	−52 ± 0.2	−53 ± 0.2
hNav 1.2	−25 ± 0.3	−43 ± 3	−61.6 ± 0.2	−66.9 ± 0.3
hNav 1.3	−20 ± 0.6	−19.9 ± 0.5	−53.9 ± 0.5	−52.6 ± 0.6
hNav 1.4	−4.2 ± 0.4	−4.4 ± 0.4	− 43.8 ± 0.3	−44.2 ± 1.4
hNav 1.5	−25.7 ± 0.3	−25 ± 0.3	−59.7 ± 0.3	60 ± 0.3
hNav 1.6	−22.8 ± 0.3	− 45.2 ± 1	−56.4 ± 0.3	−62.3 ± 0.2
hNav 1.7	−25.7 ± 0.3	−27 ± 0.4	−71.5 ± 0.4	70.6 ± 0.3

V_1/2_ = middle activation or inactivation potential

## Discussion

In Mexico, approximately 300,000 people per year are reported as having been stung by a scorpion. Clinical application of a commercially available horse antivenom has reduced the deadly cases to less than 100 persons yearly [10]. The *Centruroides hirsutipalpus* scorpion is endemic in Minatitlan, a small area in the state of Colima with 8985 inhabitans, in which 176 cases of human envenomation by this scorpion have been reorted. Thus far, no data concerning the venom composition of this scorpion species or its effects are available. This communication reports the HPLC separation of its soluble venom, the determination of the molecular weight of the main components as determined by mass spectrometry analysis, as well the electrophysiological effects of the soluble venom on various types of ion channels, known to be the target of scorpion toxins.

The physiological effects of these venom components produce an abnormal massive depolarization of the target cells causing impairment of their proper function [11]. We report herein that the venom of *C. hirsutipalpus* is a complex mixture containing at least 83 different components. Since this venom might also contain protease enzymes, it is possible that some of the peptides identified by mass spectrometry are produced by enzymatic cleavage of original peptides/proteins. However, as mentioned in Table 1, a few fractions were not identified (ND) by mass spectrometry. Usually this is due to proteins (mainly enzymes of higher molecular weight, as discussed in [12]) that cannot be identified by the mass spectrometer we used. Thus, the suggestion that this venom contains at least 80 distinct components is reasonable. The majority of peptides have a molecular weight ranging from 7001 to 8000 (46% components fall in this category) as shown in Fig. 2, which usually corresponds to peptides known to affect Na^+^-channels.

From the medical point of view, the sodium-channel-specific toxins are the most important ones. They usually are polypeptides with a length of 61–76 amino acids, folded with four disulfide bonds [13]. Particularly in this species a large number of components having a molecular mass similar to the sodium-channel-specific toxins were found. Approximately 33% of the peptides found in another phylogenetically related scorpion species occurring in Colima (*Centruroides tecomanus*) corresponded to toxins with this molecular weight [14]. In some other scorpions of the same genus, such as *Centruroides noxius*, the most poisonous not only in Mexico but also in the world, seven toxins are known to be specific to mammals [15]. The electrophysiological analysis of *C. hirsutipalpus* venom shows an effect on the channels Nav 1.1, 1.2 and 1.6. The current modification produced by this venom is described as a beta effect, which means channel activation at a more negative potential and gradual decrease of the peak current [16–18].

In addition to the sodium toxins, the molecules that are also responsible for the toxicity of the venom are peptides in the range of molecular masses from 3000 to 5000 Da, which are K^+^-channel blockers (28% of these are listed, see Table 1) [13–19]. The effect on hERG and hKv 1.1 channels is displayed in Fig. 4; the currents were reduced with soluble *Centruroides hirsutipalpus* venom.

As we stated previously, the newly sequenced peptides are very interesting given their high similarity to other known peptides isolated from the venom of various *Centruroide*s species. Peptide A of Fig. 1 is 97% identical to a peptide (CllNtx). This peptide was assayed for various possible functions, such as mouse and insect toxicity, antimicrobial activity and K^+^-channel-blocking effects, all of which were negative and presented no effects. Thus far, it is considered to be an “orphan peptide” [8], whereas peptide B in Fig. 1 is 89% identical to Cn5, an arthropod toxin found to be identical in two different scorpion species (*C. noxius* and *C. suffusus suffusus*). Cn5 is toxic to crustaceans and its three-dimensional structure was determined [20]. Thus, the results reported herein are original data that confirm similar findings in related species of Mexican scorpions.

This communication contributes to the knowledge on the toxicity of the species *Centruroides hirsutipalpus*, which may in the future improve current antivenoms by investigating targets for toxins from this species that have not yet been evaluated.

## Conclusion

In conclusion, this work reports the fingerprinting mass of components from the venom of *Centruroides hirsutipalpus*, finding a predominance of toxins specific to sodium or potassium channels, as shown in the mass fingerprinting of the soluble venom. Two new peptides were fully sequenced. One was classified as “orphan peptide” of unknown function and the other peptide as an arthropod toxin. The whole soluble venom is demonstrated to affect Nav channels 1.1, 1.2 and 1.6, and potassium channels hERG and hKv 1.1.

## Abbreviations

DMEM: Dulbecco’s modified Eagle medium
FBS: fetal bovine serum
HPLC: high performance liquid chromatography
MW: molecular weight
ND: not identified
RT: retention times
TFA: trifluoroacetic acid

## References

[1] Zhang L, Shi W, Zeng XC, Ge F, Yang M, Nie Y (2015). Unique diversity of the venom peptides from the scorpion *Androctonus bicolor* revealed by transcriptomic and proteomic analysis. J Proteome.

[2] Possani LD, Merino E, Corona M, Bolivar F, Becerril B (2000). Peptides and genes coding for scorpion toxins that affect ion-channels. Biochimie.

[3] Kouznetsov V: Kalium. Defensa química en la naturaleza. http://kaliumdb.org. Accessed 18 Jul 2017.

[4] Dehesa-Dávila M, Possani LD (1994). Scorpionism and serotherapy in Mexico. Toxicon.

[5] Ponce-Saavedra J, Francke OF (2009). Descripción de una especie nueva de alacrán con importancia médica del género *Centruroides* (Scorpiones: Buthidae) del estado de Colima. México Rev Mex Biodiv.

[6] Riaño-Umbarila L, Rodríguez-Rodríguez ER, Santibañez-López CE, Güereca L, Uribe-Romero SJ, Gómez-Ramírez IV (2017). Updating knowledge on new medically important scorpion species in Mexico. Toxicon.

[7] Olamendi-Portugal T, Restano-Cassulini R, Riaño-Umbarila L, Becerril B, Possani LD (2017). Functional and immuno-reactive characterization of a previously undescribed peptide from the venom of the scorpion *Centruroides limpidus*. Peptides.

[8] Lebreton F, Delepierre M, Ramírez AN, Balderas C, Possani LD (1994). Primary and NMR three-dimensional structure determination of a novel crustacean toxin from the venom of the scorpion *Centruroides limpidus limpidus* Karsch. Biochemistry.

[9] Cid Uribe JI, Jiménez Vargas JM, Ferreira Batista CV, Zamudio Zuñiga F, Possani LD (2017). Comparative proteomic analysis of female and male venoms from the Mexican scorpion *Centruroides limpidus*: novel components found. Toxicon.

[10] Chávez-Haro AL, Ortiz E. Scorpionism and dangerous species of Mexico.In Gopalakrishnacoke P, Possani LD, Schwartz EF, Rodriguez de la Vega RC editors. Scorpion Venoms. Dordrecht: Springer; 2015. p201-213.

[11] Catterall WA, Cestèle S, Yarov-Yarovoy V, Yu FH, Konoki K, Scheuer T (2007). Voltage-gated ion channels and gating modifier toxins. Toxicon.

[12] Batista CVF, del Pozo L, Zamudio FZ, Contreras S, Becerril B, Wanke E (2004). Proteomics of the venom from the Amazonian scorpion *Tityus cambridgei* and the role of prolines on mass spectrometry analysis of toxins. J Chromatogr B Analyt Technol Biomed Life Sci.

[13] Possani LD, Becerril B, Delepierre M, Tytgat J (1999). Scorpion toxins specific for Na+−channels. Eur J Biochem.

[14] Valdez-Velázquez LL, Quintero-Hernández V, Romero-Gutiérrez MT, Coronas FI, Possani LD (2013). Mass fingerprinting of the venom and transcriptome of venom gland of scorpion *Centruroides tecomanus*. PLoS One.

[15] Quintero-Hernández V, Jiménez-Vargas JM, Gurrola GB, Valdivia HH, Possani LD (2013). Scorpion venom components that affect ion-channels function. Toxicon.

[16] Cestèle S, Qu Y, Rogers JC, Rochat H, Scheuer T, Catterall WA (1998). Voltage sensor-trapping: enhanced activation of sodium channels by beta-scorpion toxin bound to the S3-S4 loop in domain II. Neuron.

[17] Cestèle S, Scheuer T, Mantegazza M, Rochat H, Catterall WA (2001). Neutralization of gating charges in domain II of the sodium channel alpha subunit enhances voltage-sensor trapping by a beta-scorpion toxin. J Gen Physiol.

[18] Cestèle S, Yarov-Yarovoy V, Qu Y, Sampieri F, Scheuer T, Catterall WA (2006). Structure and function of the voltage sensor of sodium channels probed by a beta-scorpion toxin. J Biol Chem.

[19] Possani,LD: Structure of scorpion toxins. In Tu AT, editor. Handbook of natural toxins, vol.2. New York: Marcel Dekker, Inc. 1984. p513-550

[20] Corzo G, Prochnicka-Chalufour A, García BI, Possani LD, Delepierre M (2009). Solution structure of Cn5, a crustacean toxin found in the venom of the scorpions *Centruroides noxius* and *Centruroides suffusus suffusus*. Biochim Biophys Acta.

